# A robust reduction in near-surface wind speed after volcanic eruptions: Implications for wind energy generation

**DOI:** 10.1016/j.xinn.2024.100734

**Published:** 2025-01-06

**Authors:** Cheng Shen, Zhi-Bo Li, Fei Liu, Hans W. Chen, Deliang Chen

**Affiliations:** 1 Regional Climate Group, Department of Earth Sciences, University of Gothenburg, 40530 Gothenburg, Sweden; 2 Laboratory for Climate and Ocean-Atmosphere Studies, Department of Atmospheric and Oceanic Sciences, School of Physics, Peking University, Beijing 100871, China; 3 School of Atmospheric Sciences, Sun Yat-Sen University, Key Laboratory of Tropical Atmosphere-Ocean System Ministry of Education, and Southern Marine Science and Engineering Guangdong Laboratory, Zhuhai 519082, China; 4 Department of Space, Earth and Environment, Chalmers University of Technology, 41258 Gothenburg, Sweden; 5 Department of Earth System Sciences, Tsinghua University, Beijing 100084, China

**Keywords:** near-surface wind speed, volcanic eruptions, wind energy, last millennium, aerosol forcing

## Abstract

Near-surface wind speed (NSWS), a determinant of wind energy, is influenced by both natural and anthropogenic factors. However, the specific impacts of volcanic eruptions on NSWS, remain unexplored. Our simulations spanning the last millennium reveal a consistent 2-year global NSWS reduction following 10 major historical eruptions. This equates to an NSWS decrease of approximately two inter-annual standard deviations from AD 851 to 1849. This reduction is linked to the weakening of subtropical descending air and a decrease in downward momentum flux, triggered by volcanic aerosol forcing. The 1815 Tambora eruption, one of the most powerful in recent history, led to a ∼9.2% reduction in global wind power density in the subsequent 2 years. Our research fills a knowledge gap, establishes a theoretical foundation for empirical studies, and highlights the potential wind energy risks linked to large atmospheric aerosol injections, including volcanic eruptions, nuclear warfare, and climate intervention.

## Public summary

• Volcanic eruptions and geoengineering aerosol injections could significantly reduce wind speeds, impacting wind energy.
• Simulations reveal a consistent 2-year global wind speed reduction after volcanic eruptions.
• The reduction is linked to a decrease in downward momentum flux, triggered by volcanic aerosol forcing.
• The 1815 Tambora eruption led to a ∼9.2% reduction in global wind power density in the following 2 years.

## Graphical abstract

**Figure:**
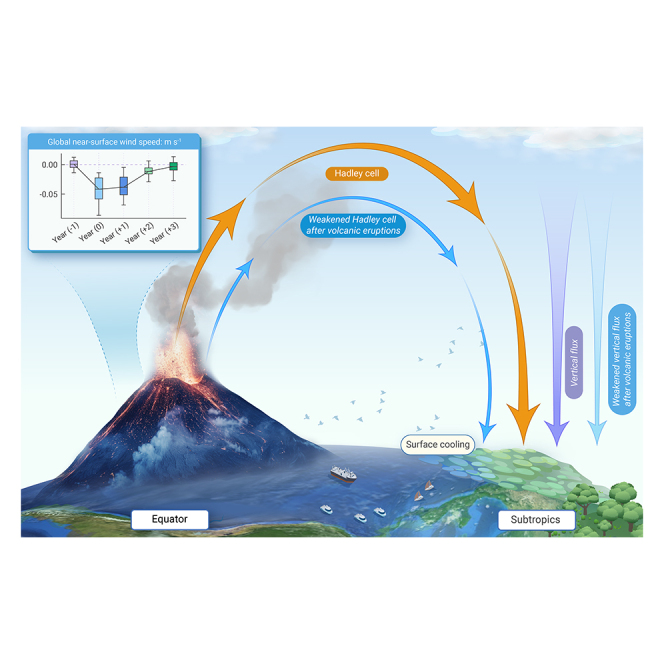

## Introduction

Near-surface wind speed (NSWS), 10 m above ground, has attracted considerable attention due to its implications for wind resource utilization, human health, and environmental governance.^1^^,^^2^^,^^3^^,^^4^ Changes in NSWS are linked to variations in atmospheric and surface conditions, such as large-scale atmospheric circulations,^1^^,^^2^^,^^5^^,^^6^^,^^7^^,^^8^^,^^9^ vegetation coverage,^10^ urbanization,^11^ anthropogenic aerosols,^12^^,^^13^ and the greenhouse effect.^14^^,^^15^ However, the global NSWS response to powerful natural external forcings, such as volcanic eruptions, remains largely unexplored.^2^ These eruptions have profound societal and environmental impacts, injecting sulfur dioxide into the stratosphere, where chemical reactions transform it into sulfate aerosols. These aerosols scatter incoming solar radiation, resulting in a negative radiative forcing on the climate system. Understanding how volcanic aerosols impact NSWS is crucial for addressing potential energy crises caused by climate interventions involving stratospheric aerosol injections, which have similar inter-annual climate effects as volcanic eruptions.^16^^,^^17^

Large volcanic eruptions significantly alter atmospheric composition, atmosphere-ocean dynamics, the hydrological cycle, and the carbon cycle.^18^^,^^19^^,^^20^^,^^21^^,^^22^ Modern observations, paleoclimate proxies, and climate model simulations offer multiple approaches for investigating volcanic forcing effects. Previous studies have found that volcanic forcing weakens the global hydrological cycle,^20^^,^^23^ strengthens the polar vortex,^24^ and intensifies the Atlantic Meridional Overturning Circulation.^25^ While some studies have revealed temperature and precipitation changes following volcanic eruptions,^26^^,^^27^ the effects and physical mechanisms of volcanic forcing on global NSWS changes have not been studied previously.^28^^,^^29^

In this study, we quantify the global NSWS response to large tropical volcanic eruptions and elucidate the underlying physical mechanisms by using last-millennium (LM) simulations. The model simulations suggest a robust reduction in global NSWS in response to the volcanic aerosol forcing, particularly in subtropical regions, in the 2 years following volcanic eruptions.

## Results

### Robust reduction in NSWS after tropical volcanic eruptions

Most *in situ* wind monitoring stations are located in Europe, East Asia, and North America, with limited coverage in other regions.^30^^,^^31^ The existing observations, covering only the last four decades, are insufficient for studying the impact of rare, strong volcanic eruptions. Thus, to better understand the global NSWS response to volcanic eruptions, we used a set of model simulations over a long period, including over 10 strong volcanic eruptions, along with a superposed epoch analysis (for details see materials and methods).^32^^,^^33^^,^^34^ This involved examining LM simulations based on multiple datasets from different sources, including 10 models from the Paleoclimate Modeling Intercomparison Project phases 3 (PMIP3) and 4 (PMIP4), and five volcanic-forcing members from the Community Earth System Model-Last Millennium Ensemble (CESM-LME), listed in Table S1. For each model, we used the 10 strongest tropical volcanic eruptions to calculate the multi-model ensemble mean for these events.

**Figure 1:**
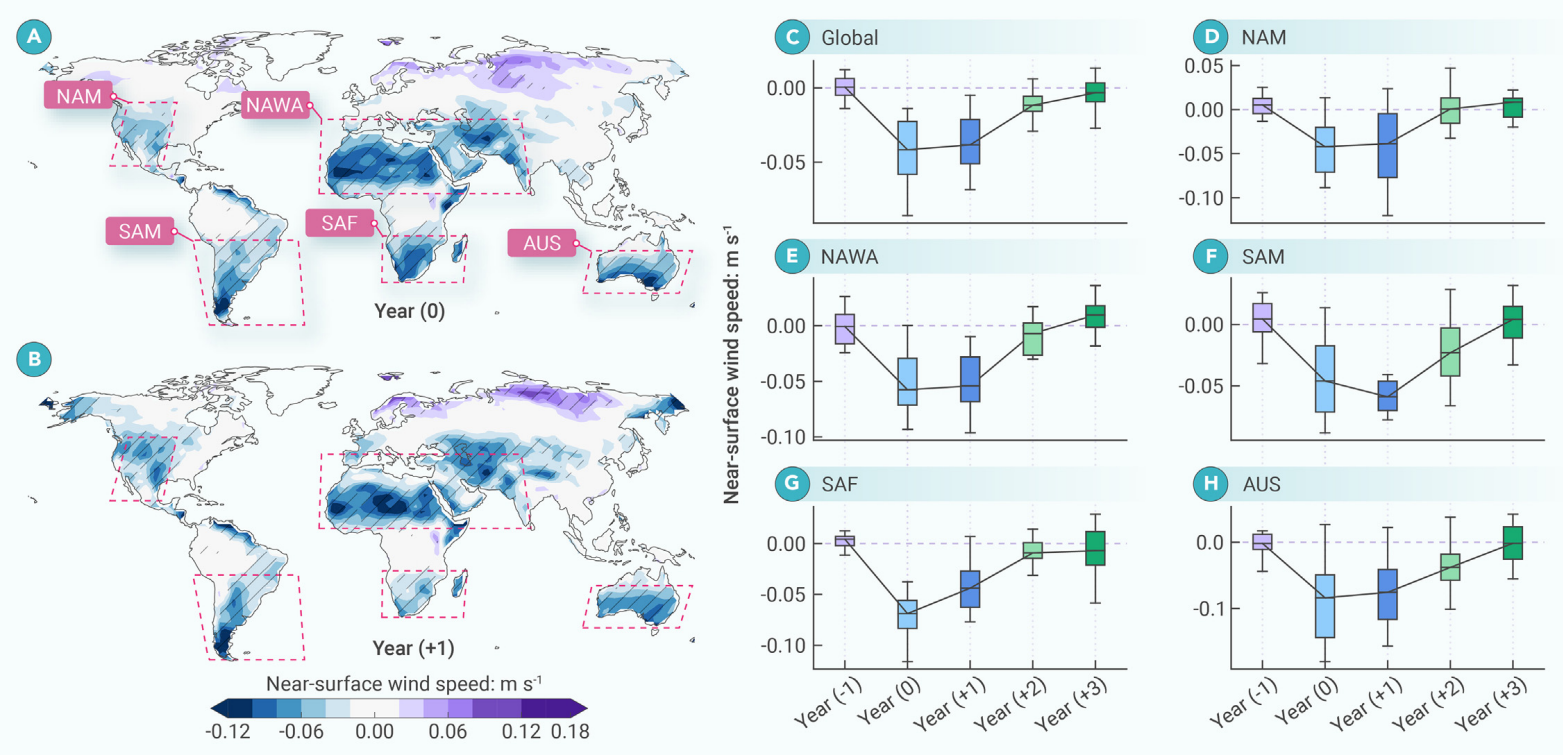
Annual-mean near-surface wind speed response to large tropical volcanic eruptions (A) Anomalous annual-mean near-surface wind speed (NSWS) (m s^−1^) in the eruption year for the average of the 10 largest tropical eruptions documented in last-millennium simulations (AD 850–1850). Hatching denotes anomalies significant at the 0.05 level. (B) Same as (A), but for responses in the first post-eruption year. (C) Globally average NSWS changes from the year before the eruption to the third post-eruption year. Year (0) denotes the eruption year. Anomalies were calculated relative to the average value during the 5 years before each eruption. The error bars denote the full range in the models and the boxes cover the 25th–75th percentiles. (D–H) Same as (C), but for area-averaged NSWS changes over North America (NAM), North Africa-West Asia (NAWA), South America (SAM), South Africa (SAF), and Australia (AUS), respectively (see Table S2 for the definition of the regions).

The spatial distributions of NSWS responses to large tropical volcanic eruptions in the eruption year (year (0)) and the first year after the eruption (year (+1)) are similar (Figures 1A and 1B), with a pattern correlation coefficient of 0.74 (*p* < 0.01). After the eruptions, the model results show a significant large-scale reduction in NSWS in large parts of the subtropics, including North America (NAM), North Africa-West Asia (NAWA), South America (SAM), South Africa (SAF), and Australia (AUS) (see Table S2 for region definitions). Some significant NSWS increases were found in small parts of the Eurasian high latitudes. The decreased subtropical NSWS dominated the global-averaged NSWS variations in year (0) and year (+1) (Figures 1C–1H). NSWS then recovered to its normal climatological state in each region, suggesting that the effects of large tropical volcanic forcing on NSWS last for approximately 2 years (Figure 1C). The spatial patterns of the volcanic effects on NSWS are highly consistent between the volcano-only and all-forcing experiments of CESM-LME (Figure S1), suggesting that the use of volcano-only forcings of the CESM-LME did not influence the robustness of our findings. Calculated reductions in NSWS during eruption years were −0.04 m s^−1^ (Global), −0.04 m s^−1^ (NAM), −0.06 m s^−1^ (NAWA), −0.05 to −0.06 m s^−1^ (SAM), −0.05 to −0.07 m s^−1^ (SAF), and −0.07 to −0.08 m s^−1^ (AUS) (Figures 1C–1H). The amplitude of the reduction is ∼2 times the inter-annual standard deviation from AD 851 to 1849, highlighting the strong disturbance in NSWS caused by tropical volcanic eruptions. As a comparison, El Niño-Southern Oscillation (ENSO) is one of the strongest factors in modulating inter-annual climate variation, and the magnitude of ENSO-induced NSWS changes in observations (Figure S2) are similar to NSWS responses to volcanic forcing shown in Figure 1. Overall, a robust decrease in NSWS was observed mainly in subtropical arid regions, except for SAM. These areas are typically characterized by descending airflows associated with the meridional Hadley cell and sparse surface vegetation.^35^

To quantify the potential loss of wind energy due to volcanic eruptions, we calculated the changes in 100-m wind power density (WPD) (materials and methods) in five geographical regions for each large tropical volcanic eruption that occurred from AD 851 to AD 1849 (Figure S3). Based on the different volcanic forcing reconstructions in the models, we identified 16 volcanic eruption events (Table S3) that primarily recorded a significant decline in WPD in year (0) and year (+1). For instance, the Tambora eruption in 1815 is estimated to have caused a ∼9.2% reduction in global WPD.

According to the distribution of onshore wind turbines in 2020,^36^ there are ∼30,000 wind turbines operating in NAWA and ∼100,000 wind turbines in NAM, while the other regions (SAM, SAF, and AUS) feature fewer wind turbines. Notably, NAWA exhibits higher efficiency due to favorable climatological wind speeds, suggesting that the impact of reduced NSWS could be significant in this region. Although NSWS over Eurasian high latitudes showed significant increases in year (0) and year (+1), there are currently no wind turbines (nor plans to build any) in that region.^36^

The LM simulations indicate a 2-year reduction in global NSWS following tropical volcanic eruptions, with significant anomalies mainly located in subtropical regions, particularly in the western and central continents. Since the results are primarily derived from model simulations, it is essential to compare these results with real-world data. We conducted the superposed epoch analysis to assess the response of NSWS to the 1982 El Chichón and 1991 Pinatubo eruptions using the Global Surface Summary of the Day dataset^24^ (Figure S4). The averaged observational results of the two eruptions (red lines) show some similarities with the model simulations (boxes). Specifically, there was a decrease in NSWS over NAM, NAWA, and SAM in year (0) and year (+1) after the volcanic eruptions. However, the results over SAF and AUS differ from the simulations. These discrepancies may be attributed to: (1) limited observational coverage in key regions shown in Figure 1, leading to significant uncertainties in quantifying NSWS changes, (2) the observed NSWS variability being heavily influenced by internal variability (Figure S2), and (3) the limitations in models in predicting NSWS.^37^^,^^38^ In the following sections, we use the model simulations to gain a mechanistic understanding of how volcanic aerosol forcing affects NSWS.

### Physical mechanisms involved in decreasing NSWS

**Figure 2:**
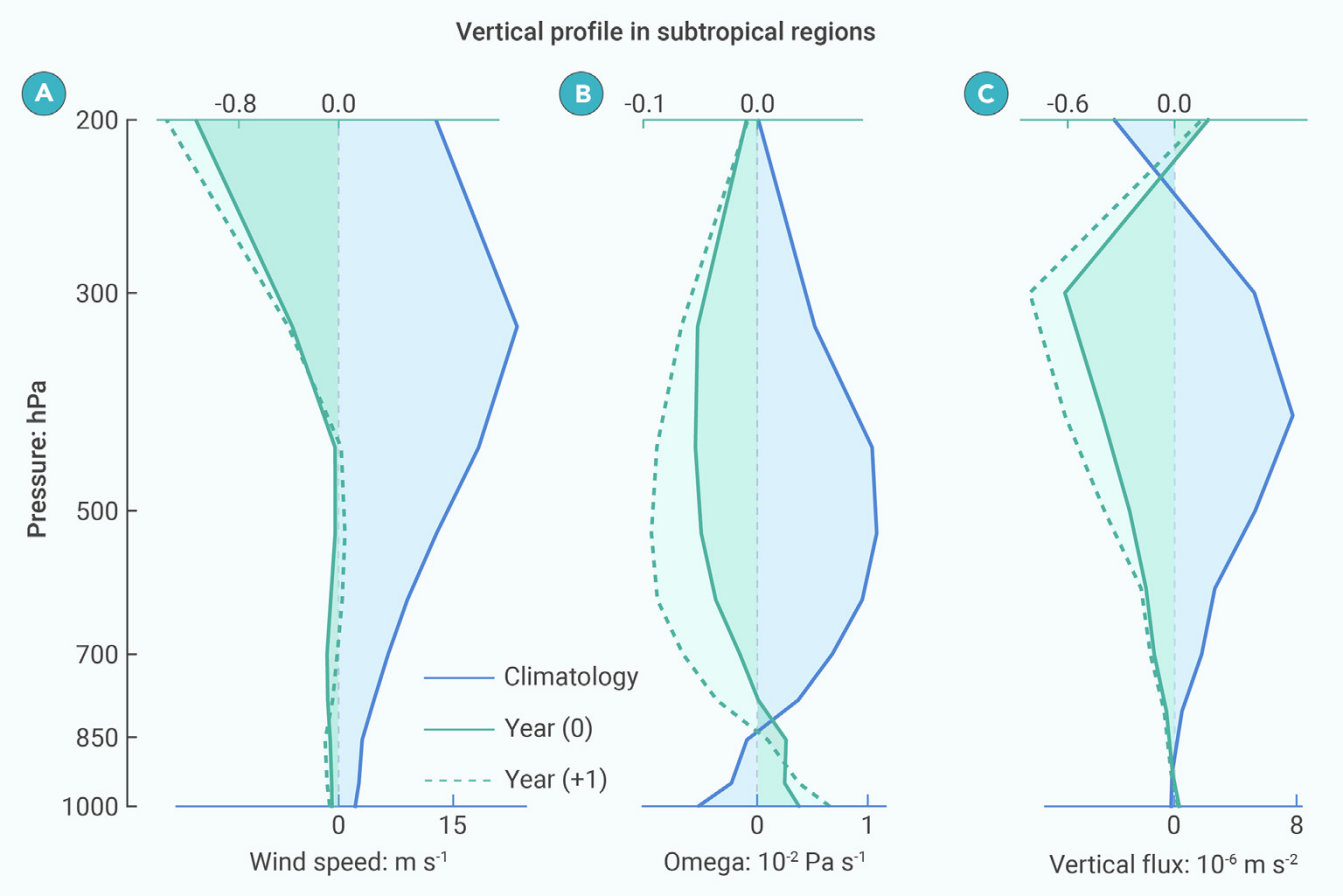
Wind speed, vertical velocity, and vertical momentum flux in response to large tropical volcanic eruptions (A) Composite of five regional (North America, North Africa*-*West Asia, South America, South Africa, and Australia) averages of the annual-mean wind speed (m s^−1^) in response to 10 large tropical volcanic eruptions based on LM simulations. Red solid, blue solid, and blue dashed lines denote the climatology (average of 5 years before the eruption), anomalies in the eruption year (year (0)), and anomalies in the first post-eruption year (year (+1)), respectively. Anomalies were calculated relative to the climatology. (B and C) Same as (A), but for vertical velocities (10^−2^ × Pa s^−1^) and vertical momentum flux (10^−6^ × m s^−2^). Positive fluxes are downward.

Vertical momentum fluxes are crucial for modulating NSWS, as winds in the upper levels of the atmosphere tend to be stronger than those near the Earth’s surface.^12^ Volcanic sulfur aerosols absorb and reflect solar radiation and absorb longwave radiation in the stratosphere, reducing surface irradiance. This leads to anomalous warming of the low-level stratosphere and surface cooling from approximately 50°S to 50°N (Figures S5A and S5B), thereby suppressing tropical convection^19^^,^^39^ and favoring weakened Hadley circulations in both hemispheres.^40^^,^^41^ The zonally averaged atmospheric overturning circulation shows weakened descending motion in the subtropics, in the descending branches of the Hadley cells, due to the weakening of the Hadley circulation (Figures S5C and S5D). Thus, for the average of the five subtropical study regions, the fastest wind speed occurs just below 300 hPa (Figure 2A), and the climatological vertical profile mainly shows a descending motion (Figure 2B). The vertical momentum fluxes (materials and methods) were mostly positive below 300 hPa in the climatological mean, indicating that momentum was transported from upper to lower levels of the troposphere in the subtropical study regions (Figure 2C).

After the tropical volcanic eruptions, reductions in horizontal wind speed primarily occurred in the upper troposphere according to models (Figure 2A). The profile of vertical velocity mainly shows an anomalous ascending motion within the troposphere, which contrasts with climatological descending flows present during non-eruption years, although with a weak descending anomaly against climatological ascending motion below 850 hPa (Figure 2B). As a result, the downward momentum flux was substantially reduced in the troposphere (Figure 2C). We also found that the horizontal momentum flux contributes negligibly to the tropospheric wind speed changes over these regions (Figure S6). We further quantified the relative contributions of vertical wind shear and vertical velocity on changes in integrated vertical momentum flux (materials and methods). Vertical wind shear made different contributions in each of the five study regions, having positive effects in NAWA and SAF, and negative effects in NAM, SAM, and AUS (Figure S7). Changes in the integrated vertical momentum flux in the 2 years following the volcanic eruptions were generally dominated by contributions from vertical velocity in all five regions (Figure S7). These results suggest that the weakened subtropical vertical circulation in response to large tropical volcanic eruptions can generate a reduced vertical momentum flux from upper levels of the atmosphere down to the surface, which weakens the NSWS (Figure 2C).

Although NSWS generally decreases over subtropical land regions after volcanic eruptions, these changes were not significant in subtropical East Asia (EA). Due to its unique topographic landscape, situated east of the Tibetan Plateau, EA is a monsoonal region dominated by climatological ascent at low levels of the troposphere.^42^^,^^43^ At the regional scale, the climatological mean wind speeds, vertical velocities, and vertical momentum fluxes were consistent between NAWA, SAF, NAM, and AUS (Figures S8–S10). In SAM and EA, the climatological mean vertical motions were ascending rather than descending (Figures S8E and F). The deviations in vertical momentum flux induced by tropical volcanic eruptions were upward in all regions, suggesting that an anomalous upward momentum flux may favor a weakened NSWS, and vice versa.

**Figure 3:**
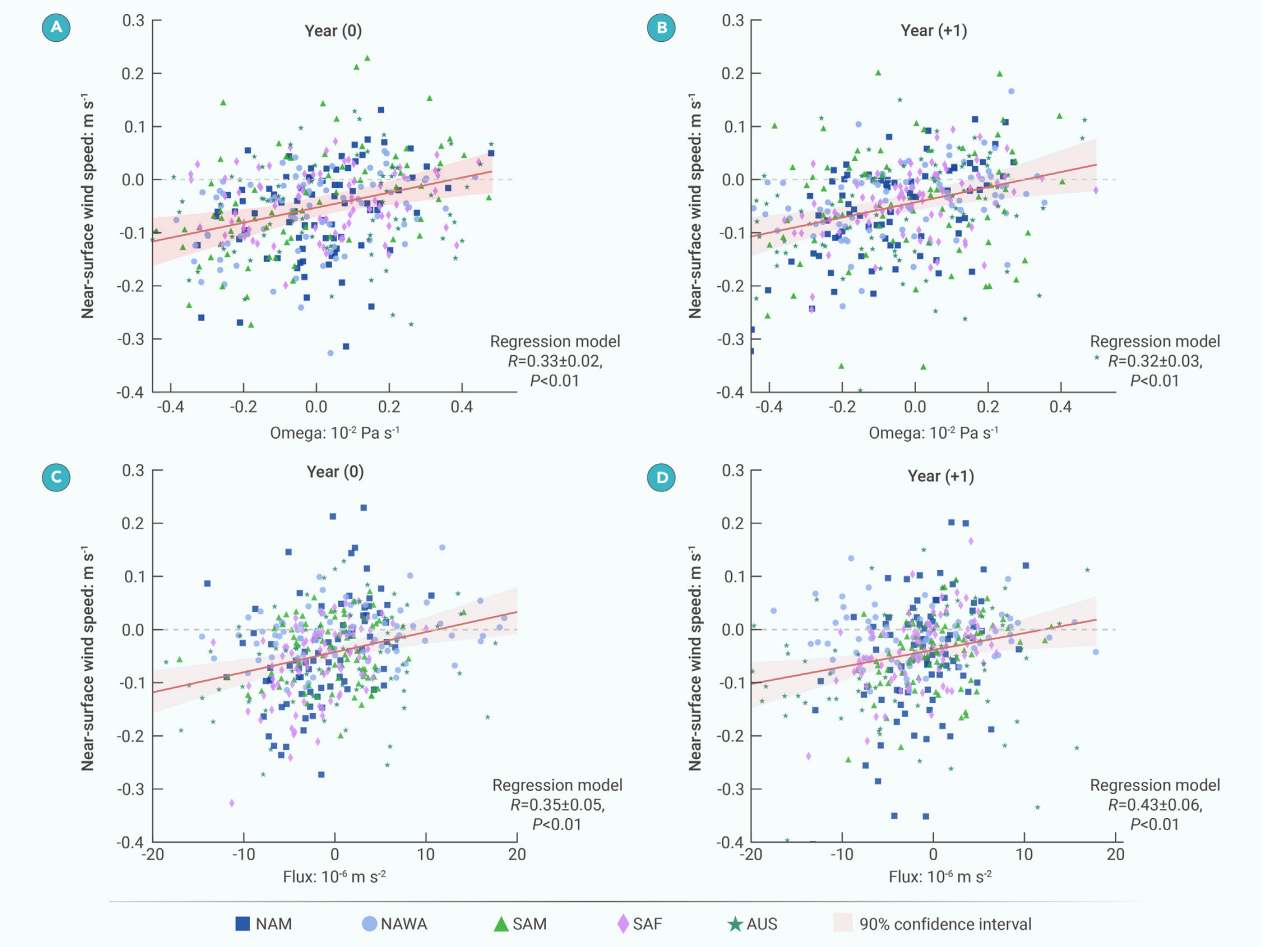
Vertical velocity and vertical momentum flux determine near-surface wind speed response to tropical volcanic eruptions (A) Regression model (materials and methods) of anomalous near-surface wind speed (m s^−1^) and 1,000–200 hPa averaged vertical velocity (10^−2^ × Pa s^−1^) in the eruption year based on LM simulations. Symbols in the legend denote the North America (NAM), North Africa-West Asia (NAWA), South America (SAM), South Africa (SAF), and Australia (AUS) regions. The red shading shows the 90% confidence interval calculated from bootstrapping. (B) Same as (A), but for the regression model in the first post-eruption year. (C and D) Same as (A and B), but for near-surface wind speed and 1,000–200 hPa integrated vertical momentum flux (10^−6^ × m s^−2^).

The relationships between NSWS and atmospheric states across the five subtropical regions were quantified by regression analysis based on model outputs (materials and methods) (Figure 3). The results show positive correlations between NSWS and averaged vertical velocity at 1,000–200 hPa (Figures 3A and 3B) in both the eruption year and the following year. We also focused on the column-integrated effect of vertical momentum flux on NSWS changes. Models show positive correlations between NSWS and the integrated vertical momentum flux at 1,000–200 hPa (Figures 3C and 3D). Although the correlation coefficients for all relationships are significant at the 0.01 level, the R^2^ values in the regressions are less than 0.2, which indicates the poor predictive power from models. The decreased downward momentum flux resulted in reduced horizontal momentum transfer from upper levels of the troposphere down to the Earth’s surface, thus offering a simple physical explanation for the decreased NSWS.

## Discussion

**Figure 4:**
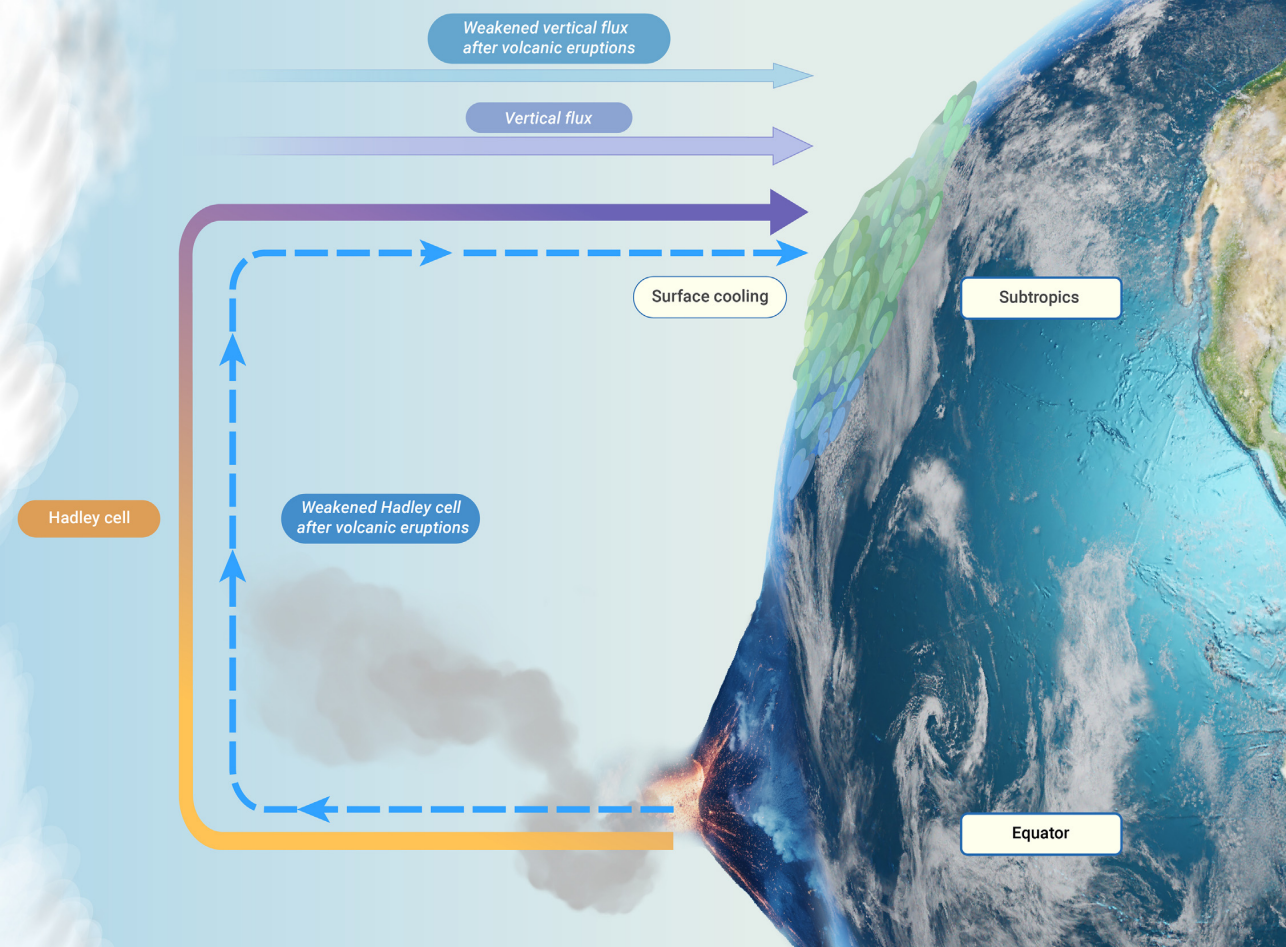
The physical mechanisms by which tropical volcanic eruptions reduce subtropical near-surface wind speed Tropical volcanic eruptions induce a weakened Hadley circulation and an anomalous ascending motion in the atmosphere over subtropical regions. A weakened vertical momentum flux from high levels of the troposphere to the Earth’s surface leads to a reduction in near-surface wind speed.

Different volcanic eruptions are associated with various component compositions including contents of CO_2_, SO_2_, Cl, and other volatiles, and also eruptive column heights, which lead to significant differences in their effects on climatic and environmental changes.^14^^,^^15^^,^^18^ In this study, we use multiple model simulations, which mainly consider the latitude-height distributions of aerosol optical depth (radiative forcing) induced by volcanic eruptions (Table S3), and the additional complexities are not considered. Model simulations suggest a 2-year weakening of global NSWS following large tropical volcanic eruptions. Figure 4 summarizes the plausible physical mechanism behind the aerosol effects of large tropical volcanic eruptions on subtropical NSWS. Aerosol forcing from these eruptions leads to global tropospheric cooling, which weakens the Hadley circulation.^40^^,^^41^ The decrease in NSWS is linked to a weakened subtropical atmospheric descent, favoring a reduced downward vertical momentum flux. Specifically, less kinetic energy is transferred from the upper levels of the troposphere to the Earth’s surface, resulting in a reduction in NSWS. We highlight that the Hadley circulation as a plausible link between the vertical momentum flux from the upper atmosphere (free troposphere) to the surface (boundary layer). Considering the comparison between model simulations and observations are not well matched, there are two key limitations in our work. First, more reliable observational data are needed in the future to verify the model results. Second, the models' ability to simulate the variability of NSWS should be further improved and studied.

Strong tropical volcanic eruptions can significantly reduce wind power density. North America and Australia have already begun to heavily utilize wind energy,^44^ and the desert regions of North Africa are considered to contain some of the world’s richest wind energy resources.^45^ In some subtropical regions, a portfolio with diverse energy sources should be carefully designed to account for the potential negative impacts of unforeseen volcanic eruptions, as well as similar effects caused by nuclear war or climate intervention. Such considerations are crucial for future planning, especially regarding wind energy production.

## Materials and methods

### LM simulations

Eleven LM climate simulations were used in this study (Table S1), including seven models from PMIP3, three models from PMIP4, and simulations from CESM-LME. Four volcanic forcing reconstructions were used in these simulations: GRA08,^46^ Ammann,^47^ CU13,^48^ and EVA(2k).^49^ Each PMIP simulation had 1 member, and the CESM-LME had 13 all forcing members and 5 volcanic forcing members. The ensemble mean of all volcanic forcing members in CESM-LME was calculated to ensure that each model had the same composite weight. All models shared the same millennium period (AD 851–1849).

For historical volcanic eruptions, different volcanic forcing reconstructions exist and they indicate different eruption dates and strengths. Following the literature,^33^^,^^50^ we formed three groups that were each comprised of the 10 largest tropical volcanic eruptions in each simulation according to GRA08,^46^ Ammann,^47^ CU13,^48^ and EVA(2k).^49^ A tropical eruption was defined by the aerosol density and aerosol optical depth being evenly distributed in both hemispheres.^46^ Considering some dating errors among the three groups, we identified 16 volcanic eruption events in 11 models from AD 850 to 1849 (i.e., AD 971, 1108, 1171, 1213, 1229/1230, 1257, 1275, 1284/1286, 1452/1456/1458, 1600, 1641, 1674, 1695/1696, 1809, 1815/1816, and 1835). The weakest eruption in this set was still stronger than the 1991 Pinatubo eruption, which indicates that a stronger response of the NSWS to volcanic forcing could be detected in LM simulations before AD 1850.

### Superposed epoch analysis and significance

Classical superposed epoch analysis^32^ was used to investigate the impact of volcanic eruptions on NSWS changes and related physical mechanisms. The effects of background noises were reduced by removing the climatology of the 5-year means that preceded each eruption. Consequently, performing the superposed epoch analysis removed most of the influence of internal variability among different models. Each model was bi-linearly interpolated into a 2° × 2° resolution before calculating the multi-model ensemble mean.^51^ Student's t test was used to test the statistical significance, assuming that each model is an independent sample.

### Wind power density

Vertical extrapolation was used to calculate wind speeds at an altitude of 100 m, which is close to the height of wind turbines, according to the power law:^52^

(Equation 1) $$U_{100m}=U{\left(\frac{H}{10}\right)}^{\alpha }$$

where $U_{100m}$ and U denote the 100- and 10-m wind speeds, respectively; α denotes the wind-shear exponent, which was a constant of value 0.14 in this study^53^; and H is the target height (100 m).

WPD was calculated as the wind power per unit area^54^:

(Equation 2) $$WPD=\frac{1}{2}\rho {U_{100m}}^{3}$$

Here, we assumed that the air density was globally uniform^55^ $\left(\rho =1.225\;{\text{kg}}^{-1}m^{-3}\right)$.

## Momentum flux transport

The temporal evolutions of wind speed can be written as:

(Equation 3) $$WS=\sqrt{u^{2}+v^{2}}$$

(Equation 4) $$\frac{\partial WS}{\partial t}\propto -u\cdot \frac{\partial WS}{\partial x}-v\cdot \frac{\partial WS}{\partial y}-\omega \cdot \frac{\partial WS}{\partial P}$$

where *u* and *v* denote the zonal and meridional wind speeds, respectively; ω denotes the vertical velocity in an isobaric coordinate system; and *WS* denotes total horizontal wind speed.

Then, the horizontal and vertical momentum flux were defined as:

(Equation 5) $$\text{horizontal}\;\text{flux}=-u\cdot \frac{\partial WS}{\partial x}-v\cdot \frac{\partial WS}{\partial y}$$

(Equation 6) $$\text{vertical}\;\text{flux}=-\omega \cdot \frac{\partial WS}{\partial P}$$

The relative contributions of vertical velocity and vertical wind shear to changes in the vertical flux of horizontal momentum were determined by deconstructing Eq. 6 as follows:

(Equation 7) $$\Delta \left(\omega \cdot \frac{\partial WS}{\partial P}\right)=\Delta \omega \cdot \frac{\partial \bar{WS}}{\partial P}+\bar{\omega }\cdot \Delta \frac{\partial WS}{\partial P}+\Delta \omega \cdot \Delta \frac{\partial WS}{\partial P}$$

where Δ and the overbar denote anomaly and climatology, respectively. The three terms on the right-hand side represent the contributions from vertical velocity, vertical wind shear, and nonlinear processes, respectively.

### Linear regression analysis

Linear regression was performed on the LM simulation outputs using the least-squares method to identify the general relationships between NSWS and other physical variables. To assess the uncertainty of these relationships, we employed a bootstrap method, which is a commonly used statistical technique for estimating confidence intervals and testing hypotheses.^56^^,^^57^^,^^58^ The 90% confidence interval was calculated using the bootstrap method^56^ as follows:

(1) Ten thousand bootstrap samples were created from the dataset.
(2) The statistics (e.g., mean, median, standard deviation) were calculated for each bootstrap sample.
(3) The 5th and 95th values in the sorted list corresponded to the 5th and 95th percentiles of the statistical distribution, respectively. The interval between these two values was the 90% confidence interval for the true value of the statistics.

## Acknowledgments

The research presented in this article is a contribution to the strategic research area Modeling the Regional and Global Earth system, MERGE. This work was supported by Swedish Formas (2019-01520 and 2023-01648), the 10.13039/501100001809Natural Science Foundation of China (42488201 and 41975107), and the Guangdong Major Project of Basic and Applied Basic Research (2020B0301030004). C.S. is also supported by the Sven Lindqvists Forskningsstiftelse, 10.13039/501100008584Stiftelsen Längmanska Kulturfonden (BA24-0484), Stiftelsen Åforsk (24-707) and Adlerbertska Forskningsstiftelsen (AF2024-0069). This paper is WePre publication 002.

## Author contributions

Z.-B.L., D.C., and C.S. designed the research. C.S. and Z.-B.L. analyzed data, performed figures, and wrote the original draft. All authors reviewed and revised the manuscript.

## Declaration of interests

The authors declare no competing interests.

## Lead contact website

https://www.gu.se/en/about/find-staff/zhi-boli-0.

## Supplemental information

Document S1. Figures S1–S10 and Tables S1–S3

Document S2. Article plus supplemental information
