# Supplementary material for: A robust reduction in near-surface wind speed after volcanic eruptions: Implications for wind energy generation
Source: Innovation (Camb). 2025 Jan 6;6(1):100734. doi: 10.1016/j.xinn.2024.100734 (PMC11763914; doi:10.1016/j.xinn.2024.100734)
Supplement: Document S1. Figures S1–S10 and Tables S1–S3 [file mmc1.pdf]

The Innovation, Volume *6*

Supplemental Information

A robust reduction in near-surface wind speed after volcanic eruptions: Implications for wind energy generation

Cheng Shen, Zhi-Bo Li, Fei Liu, Hans W. Chen, and Deliang Chen

Supplemental Information

A robust reduction in near-surface wind speed after volcanic eruptions: implications for wind energy generation

Cheng Shen, Zhi-Bo Li, Fei Liu, Hans W. Chen, Deliang Chen

Table of Contents

Table. S1. The 11 last-millennium simulations used in this study. Basic information for the seven PMIP3 models, three PMIP4 models, and CESM-LME. GRA08, Ammann, CU13, and EVA(2k) are the four volcanic forcing reconstructions used in the model simulations.

Table. S2. Spatial extent of all six study regions. Longitude and latitude ranges for all six study regions: North America (NAM), North Africa–West Asia (NAWA), South America (SAM), South Africa (SAF), Australia (AUS), and East Asia (EA).

Table. S3. Largest tropical volcanic eruptions during 850–1850 AD used in the three volcanic forcing reconstructions. Each group contained ten volcanic eruption events. Aerosol optical density (AOD) was used to quantify the strength of the volcanic eruptions. The superscript "a" denotes the dates are based on top-of-atmosphere shortwave radiation in the IPSL model.

Fig. S1. Annual-mean near-surface wind speed response to large tropical volcanic eruptions in CESM-LME. (A) Anomalous annual-mean near-surface wind speed (m s^{-1}) in the eruption year for the average of the ten largest tropical eruptions documented in the CESM-LME all forcing simulations (850–1850 AD). Hatching denotes anomalies significant at the 0.05 level. (B) Same as (A), but for responses in the first post-eruption year. (C-D) Same as (A-B), but for CESM-LME volcano-only forcing simulations (850–1850 AD).

Fig. S2. ENSO-induced magnitudes of near-surface wind speed (NSWS) in observations. Niño 3.4 index regressed on the observed NSWS during 1978-2017, with black circles indicating the regression c oefficients are significant at 0.05 level. Niño 3.4 index is defined as the winter (December-January-Feb ruary) average of sea surface temperature over 5S-5N, 170W-120W from HadlSST (https://www.metof fice.gov.uk/hadobs/hadisst/data/download.html). Observed NSWS is from the Global Surface Summary of the Day dataset.

Fig. S3. Annual-mean 100-m wind power density response to large volcanic eruptions. (A) Percentage changes (%) of 100-m wind power density over North America (NAM) in the eruption year (blue) and first post-eruption year (red) for 16 volcanic eruption events based on last-millennium simulations. Error bars denote the full range of the 11 models. (B–E) Same as (A), but for changes in

North Africa–West Asia (NAWA), South America (SAM), South Africa (SAF), and Australia (AUS), respectively.

Fig. S4. Comparison between last-millennium simulations and observations. (A) Distributions of GSOD in situ stations (red dots) and key regions (purple shadings) where the anomalous near-surface wind speed (NSWS) was significant in both the eruption year (year (0)) and the first post-eruption year (year (+1)) based on the last-millennium simulations. Blue boxes denote five subtropical regions: North America (NAM), North Africa–West Asia (NAWA), South America (SAM), South Africa (SAF), and Australia (AUS). (B) NAM-averaged NSWS anomalies (m s−1) in year (0) and year (+1). The anomaly was calculated by the superposed epoch analysis. Box plots represent results from the last-millennium simulations, which are the same as that in Fig. 1. The red line represents averaged values based on the 1982 El Chichón and 1991 Pinatubo eruptions from the GSOD dataset. (C–F) Same as (B), but for NAWA, SAM, SAF, and AUS, respectively.

Fig. S5. Temperature and meridional overturning circulation responses to large tropical volcanic eruptions. (A) Climatology (five-year average before the eruption) of annual-mean zonal-averaged temperature, shown as contours (°C) and anomalous temperature in the eruption year, shown as shadings (°C) based on the last-millennium simulations. (B) Same as (A), but for responses in the first post-eruption year. (C–D) Same as (A–B), but for climatology (contours) and anomalous (shadings) mass stream function ($10^9 \times$ kg s^{-1}).

Fig. S6. Horizontal and vertical momentum flux in response to large tropical volcanic eruptions. (A) Composite of five regional (North America, North Africa–West Asia, South America, South Africa, and Australia) averages of the annual-mean horizontal momentum flux ($10^{-6} \times$ m s^{-2}) in response to ten large tropical volcanic eruptions based on LM simulations. Red solid, blue solid, and blue dashed lines denote the climatology (average of five years before the eruption), anomalies in the eruption year (year (0)), and anomalies in the first post-eruption year (year (+1)), respectively. Anomalies were calculated relative to the climatology. Positive fluxes are downward. (B) Same as (A), but for vertical momentum flux ($10^{-6} \times$ m s^{-2}).

Fig. S7. Decomposition of the integrated vertical flux of horizontal momentum. (A) Contributions from vertical velocity ($\Delta\omega \cdot \frac{\partial \overline{WS}}{\partial P}$), vertical wind shear ($\overline{\omega} \cdot \Delta\frac{\partial WS}{\partial P}$), and nonlinear processes ($\Delta\omega \cdot \Delta\frac{\partial WS}{\partial P}$) in the eruption year based on last-millennium simulations. Blue, orange, magenta, green, and light brown columns represent results in North America (NAM), North Africa–West Asia (NAWA), South America (SAM), South Africa (SAF), and Australia (AUS), respectively. (B) Same as (A), but for changes in the first post-eruption year.

Fig. S8. Vertical velocity over six subtropical regions in response to large tropical volcanic eruptions. (A) Annual-mean area-averaged vertical velocity ($10^{-2} \times$ Pa s^{-1}) over North America (NAM) based on last-millennium simulations. Red solid, blue solid, and blue dotted lines denote climatology (five-year average before the eruption), anomalies in the eruption year, and anomalies in the first post-eruption year, respectively. (B–F) Same as (A), but for South Africa (SAF), North Africa–West Asia (NAWA), Australia (AUS), South America (SAM), and East Asia (EA) respectively.

Fig. S9. Wind speed over six subtropical regions in response to large tropical volcanic eruptions. (A) Annual-mean area-averaged wind speed (m s^{-1}) over North America (NAM) based on last-millennium simulations. Red solid, blue solid, and blue dotted lines denote climatology (five-year average before the eruption), anomalies in the eruption year, and anomalies in the first post-eruption year, respectively. (B–F) Same as (A), but for South Africa (SAF), North Africa–West Asia (NAWA), Australia (AUS), South America (SAM), and East Asia (EA) respectively.

Fig. S10. Vertical flux of horizontal momentum over six subtropical regions in response to large tropical volcanic eruptions. (A) Annual-mean area-averaged vertical flux of horizontal momentum ($10^6 \times$ m s^{-2}) over North America (NAM) based on last-millennium simulations. Red solid, blue solid, and blue dotted lines denote climatology (five-year average before the eruption), anomalies in the eruption year, and anomalies in the first post-eruption year, respectively. (B–F) Same as (A), but for South Africa (SAF), North Africa–West Asia (NAWA), Australia (AUS), South America (SAM), and East Asia (EA) respectively.

Table. S1. The 11 last-millennium simulations used in this study. Basic information for the seven PMIP3 models, three PMIP4 models, and CESM-LME. GRA08, Ammann, CU13, and EVA(2k) are the four volcanic forcing reconstructions used in the model simulations.

Model	Volcanic forcing	Grid cells (longitude × latitude)	Number of members	Period
**PMIP3**				
BCC-CSM1.1	GRA08	128 × 64	1	850–2000
CSIRO-Mk3L	CU13	64 × 56	1	851–1850
GISS-E2-R	CU13	144 × 90	1	850–1850
IPSL-CM5A-LR	Ammann	96 × 96	1	850–1850
MIROC-ESM	CU13	128 × 64	1	850–1849
MPI-ESM-P	CU13	192 × 96	1	850–1849
MRI-CGCM3	GRA08	320 × 160	1	850–1850
**PMIP4**				
MIROC	EVA(2k)	128 × 64	1	850–1849
MPI-ESM	EVA(2k)	192 × 96	1	850–1850
MRI-ESM2-0	EVA(2k)	320 × 160	1	850–1849
**Others**				
CESM-LME	GRA08	144 × 96	5	850–2005

Table. S2. Spatial extent of all six study regions. Longitude and latitude ranges for all six study regions: North America (NAM), North Africa–West Asia (NAWA), South America (SAM), South Africa (SAF), Australia (AUS), and East Asia (EA).

**Region**	**Range**
North America (NAM)	20° N–50° N, 120° W–95° W
North Africa–West Asia (NAWA)	10° N–40° N, 20° W–80° E
South America (SAM)	55° S–15° S, 80° W–30° W
South Africa (SAF)	35° S–10° S, 10° E–50° E
Australia (AUS)	40° S–20° S, 110° E–160° E
East Asia (EA)	10° N–40° N, 90° W–125° E

Table. S3. Largest tropical volcanic eruptions during 850–1850 AD used in the three volcanic forcing reconstructions. Each group contained ten volcanic eruption events. Aerosol optical density (AOD) was used to quantify the strength of the volcanic eruptions. The superscript “a” denotes the dates are based on top-of-atmosphere shortwave radiation in the IPSL model (Ammann et al. 2007).

**GRA08**		**CU13**		**EVA(2k)**	
**Maximum eruption year**	**Annual mean AOD**	**Maximum eruption year**	**Annual mean AOD**	**Maximum eruption year**	**Annual mean AOD**
1213	0.24	971	0.12	1108	0.13
1257	1.03	1229	0.25	1171	0.12
1275 (1278[a])	0.31	1257	0.56	1230	0.16
1284 (1286[a])	0.23	1286	0.18	1257	0.40
1452	0.61	1456	0.27	1458	0.22
1600	0.24	1674	0.13	1600	0.13
1641	0.25	1696	0.17	1641	0.12
1809	0.22	1809	0.20	1695	0.11
1815	0.44	1816	0.36	1809	0.13
1835	0.19	1835	0.13	1815	0.19

Fig. S1.

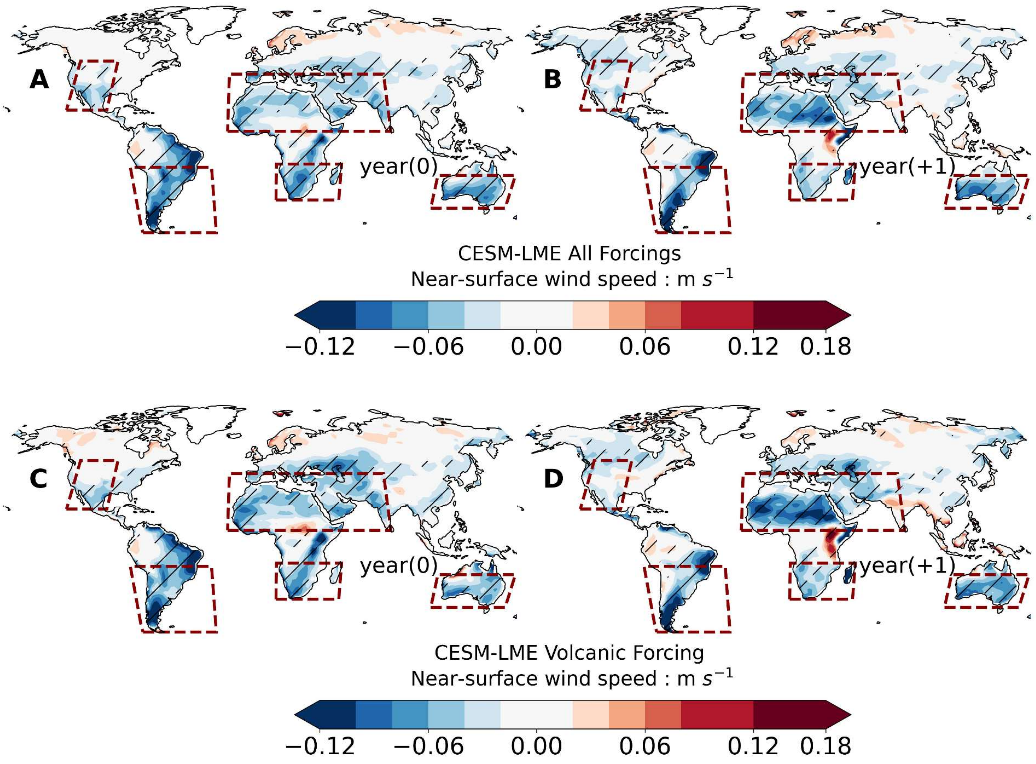

Annual-mean near-surface wind speed response to large tropical volcanic eruptions in CESM-LME. (A) Anomalous annual-mean near-surface wind speed (m s^{-1}) in the eruption year for the average of the ten largest tropical eruptions documented in the CESM-LME all forcing simulations (850–1850 AD). Hatching denotes anomalies significant at the 0.05 level. (B) Same as (A), but for responses in the first post-eruption year. (C-D) Same as (A-B), but for CESM-LME volcano-only forcing simulations (850–1850 AD).

Fig. S2.

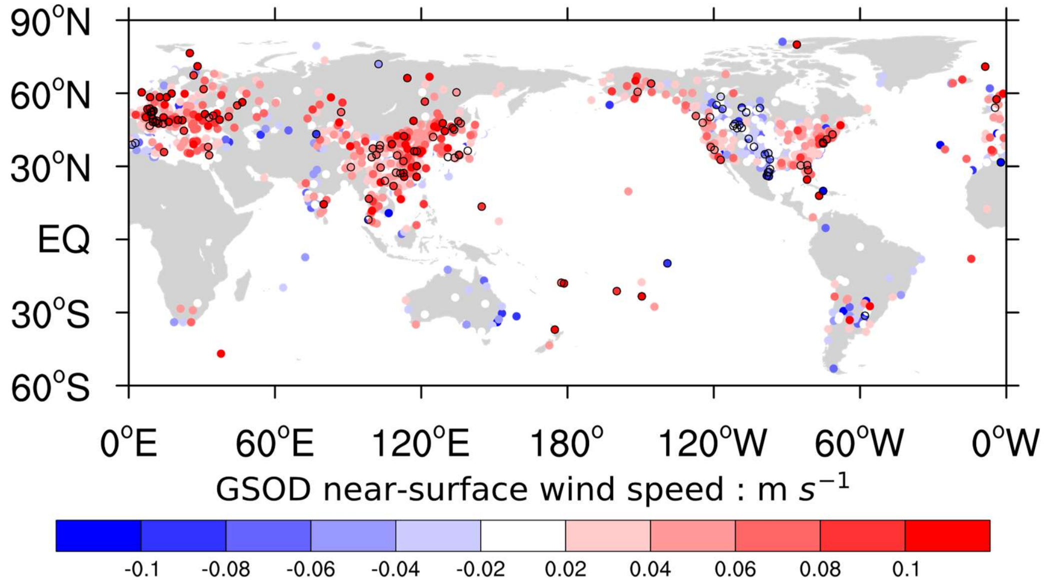

ENSO-induced magnitudes of near-surface wind speed (NSWS) in observations. Niño 3.4 index regressed on the observed NSWS during 1978-2017, with black circles indicating the regression coefficients are significant at 0.05 level. Niño 3.4 index is de fined as the winter (December-January-February) average of sea surface temperature o ver 5S-5N, 170W-120W from HadlSST (https://www.metoffice.gov.uk/hadobs/hadiss t/data/download.html). Observed NSWS is from the Global Surface Summary of the D ay dataset.

Fig. S3.

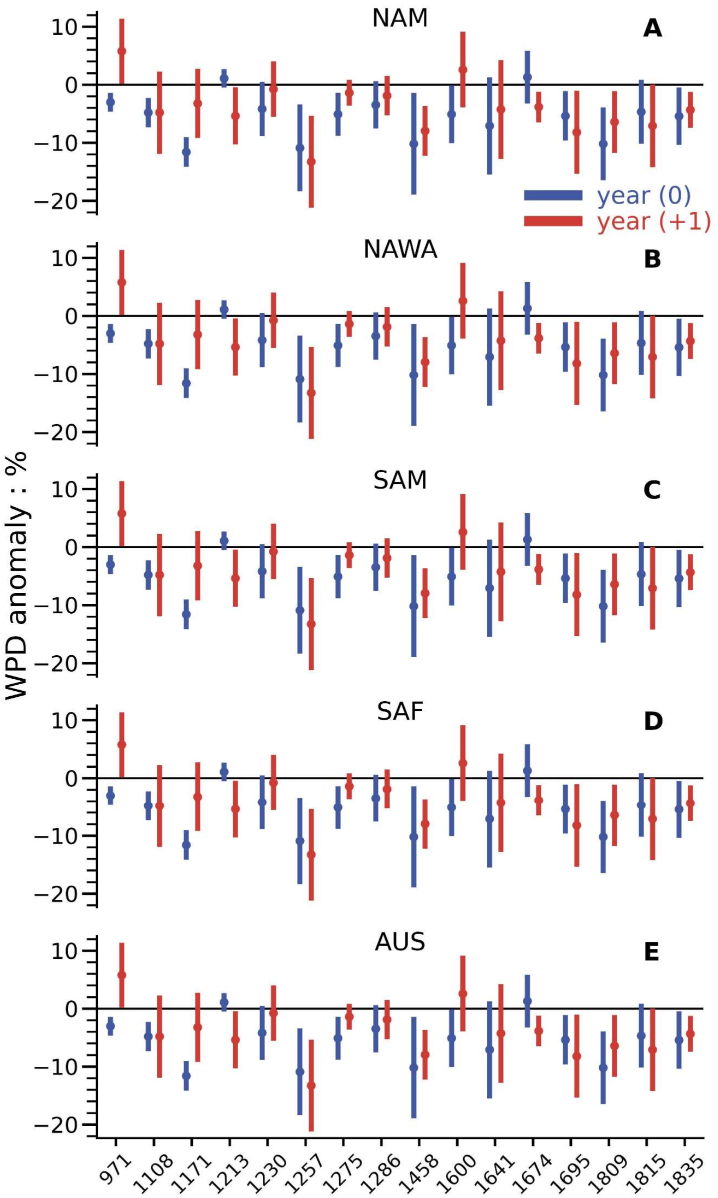

Annual-mean 100-m wind power density response to large volcanic eruptions. (A) Percentage changes (%) of 100-m wind power density over North America (NAM) in the eruption year (blue) and first post-eruption year (red) for 16 volcanic eruption events based on last-millennium simulations. Error bars denote the full range of the 11 models. (B–E) Same as (A), but for changes in North Africa–West Asia (NAWA), South America (SAM), South Africa (SAF), and Australia (AUS), respectively.

Fig. S4.

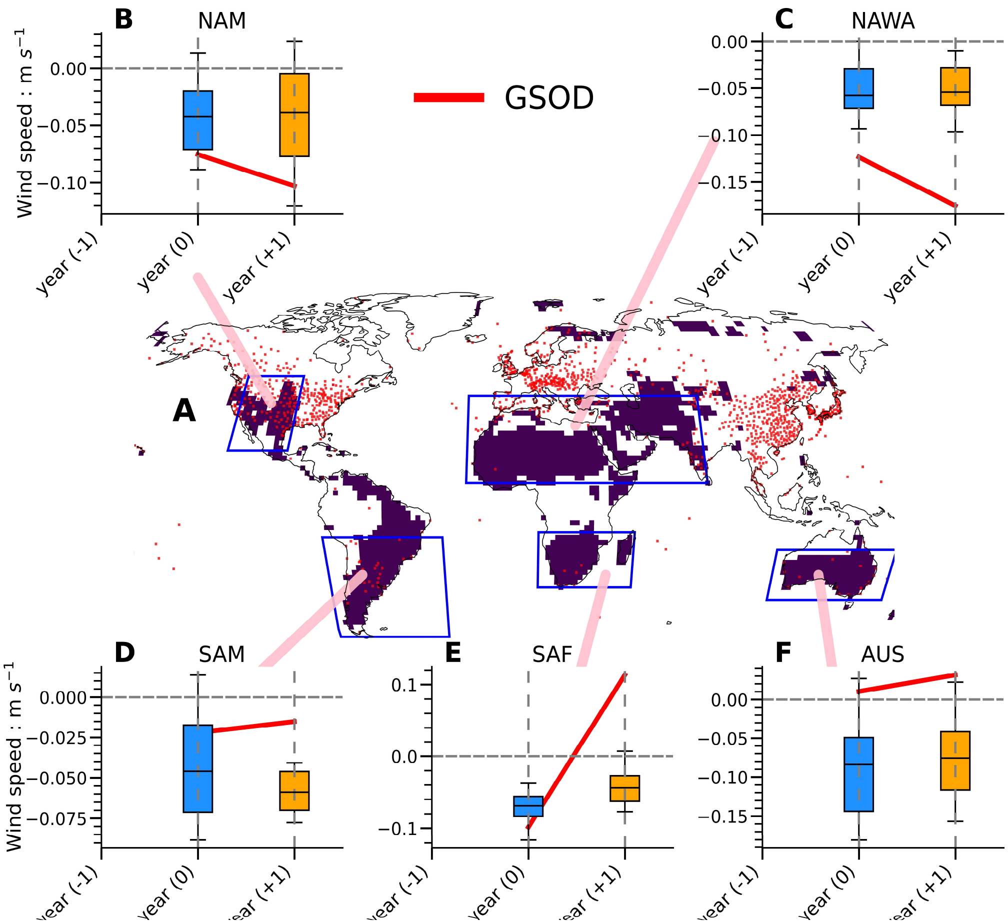

Comparison between last-millennium simulations and observations. (A) Distributions of GSOD in situ stations (red dots) and key regions (purple shadings) where the anomalous near-surface wind speed (NSWS) was significant in both the eruption year (year (0)) and the first post-eruption year (year (+1)) based on the last-millennium simulations. Blue boxes denote five subtropical regions: North America (NAM), North Africa–West Asia (NAWA), South America (SAM), South Africa (SAF), and Australia (AUS). (B) NAM-averaged NSWS anomalies (m s−1) in year (0) and year (+1). The anomaly was calculated by the superposed epoch analysis. Box plots represent results from the last-millennium simulations, which are the same as that in Fig. 1. The red line represents averaged values based on the 1982 El Chichón and 1991

Pinatubo eruptions from the GSOD dataset. (C–F) Same as (B), but for NAWA, SAM, SAF, and AUS, respectively.

Fig. S5.

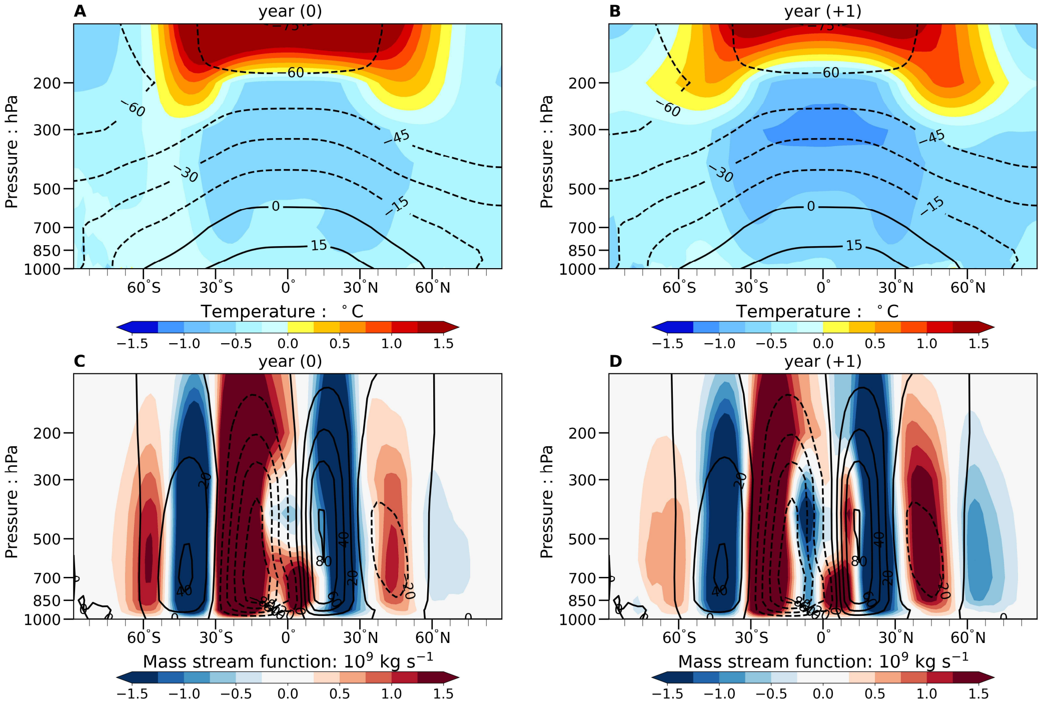

Temperature and meridional overturning circulation responses to large tropical volcanic eruptions. (A) Climatology (five-year average before the eruption) of annual-mean zonal-averaged temperature, shown as contours (°C) and anomalous temperature in the eruption year, shown as shadings (°C) based on the last-millennium simulations. (B) Same as (A), but for responses in the first post-eruption year. (C–D) Same as (A–B), but for climatology (contours) and anomalous (shadings) mass stream function (10^9 × kg s^{-1}).

Fig. S6.

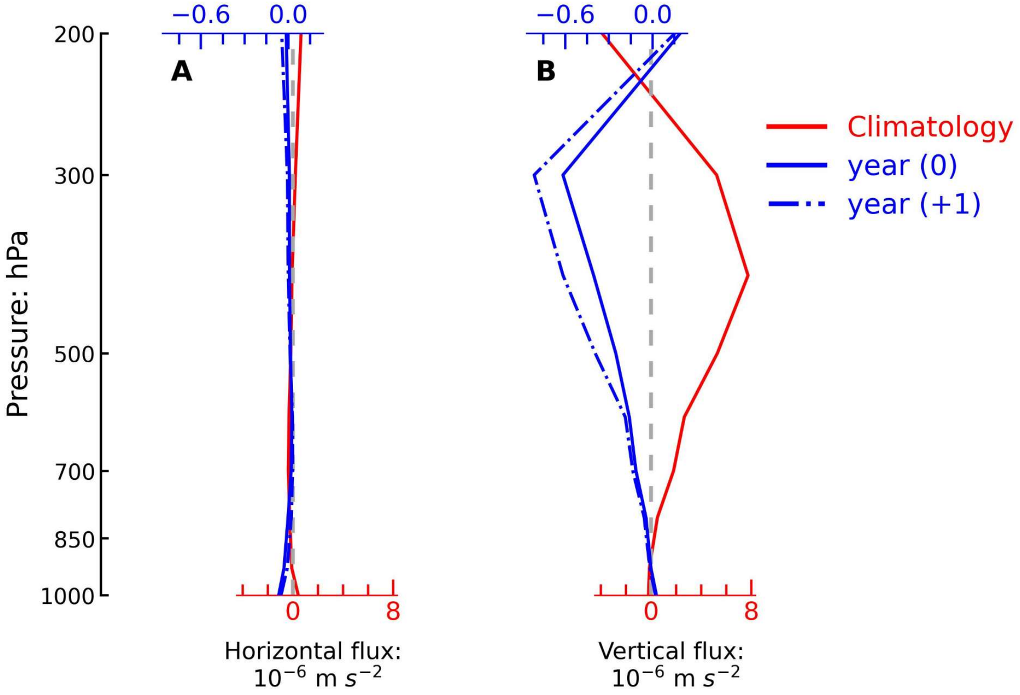

Horizontal and vertical momentum flux in response to large tropical volcanic eruptions. (A) Composite of five regional (North America, North Africa–West Asia, South America, South Africa, and Australia) averages of the annual-mean horizontal momentum flux ($10^{-6} \times$ m s^{-2}) in response to ten large tropical volcanic eruptions based on LM simulations. Red solid, blue solid, and blue dashed lines denote the climatology (average of five years before the eruption), anomalies in the eruption year (year (0)), and anomalies in the first post-eruption year (year (+1)), respectively. Anomalies were calculated relative to the climatology. Positive fluxes are downward. (B) Same as (A), but for vertical momentum flux ($10^{-6} \times$ m s^{-2}).

Fig. S7.

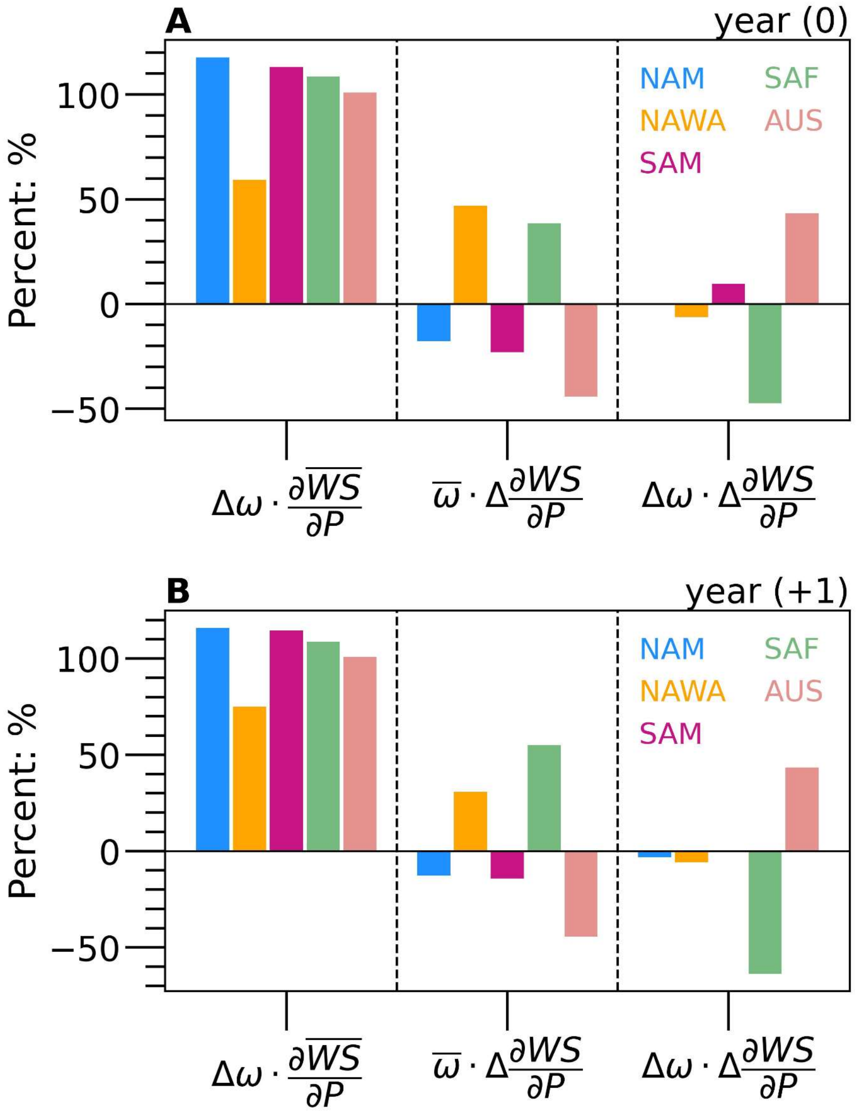

Decomposition of the integrated vertical flux of horizontal momentum. (A) Contributions from vertical velocity ($\Delta\omega \cdot \frac{\partial \overline{WS}}{\partial P}$), vertical wind shear ($\overline{\omega} \cdot \Delta\frac{\partial WS}{\partial P}$), and nonlinear processes ($\Delta\omega \cdot \Delta\frac{\partial WS}{\partial P}$) in the eruption year based on last-millennium simulations. Blue, orange, magenta, green, and light brown columns represent results in North America (NAM), North Africa–West Asia (NAWA), South America (SAM), South Africa (SAF), and Australia (AUS), respectively. (B) Same as (A), but for changes in the first post-eruption year.

Fig. S8.

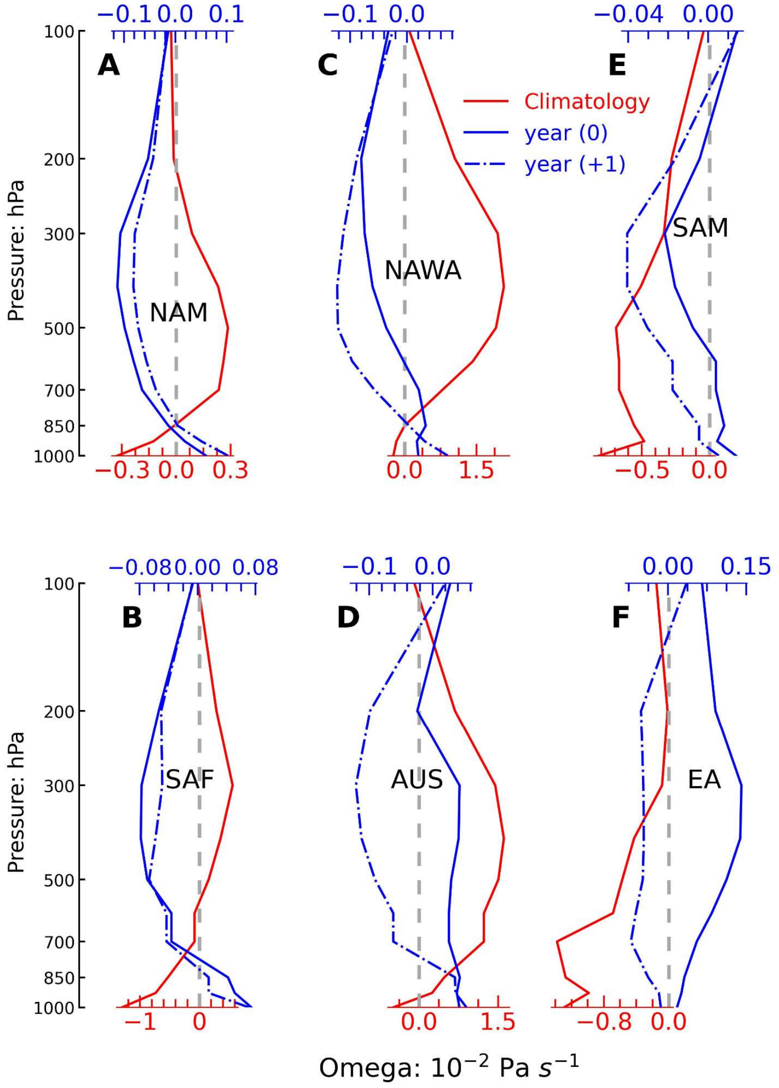

Vertical velocity over six subtropical regions in response to large tropical volcanic eruptions. (A) Annual-mean area-averaged vertical velocity ($10^{-2} \times$ Pa s^{-1}) over North America (NAM) based on last-millennium simulations. Red solid, blue solid, and blue dotted lines denote climatology (five-year average before the eruption), anomalies in the eruption year, and anomalies in the first post-eruption year, respectively. (B–F) Same as (A), but for South Africa (SAF), North Africa–West Asia (NAWA), Australia (AUS), South America (SAM), and East Asia (EA) respectively.

Fig. S9.

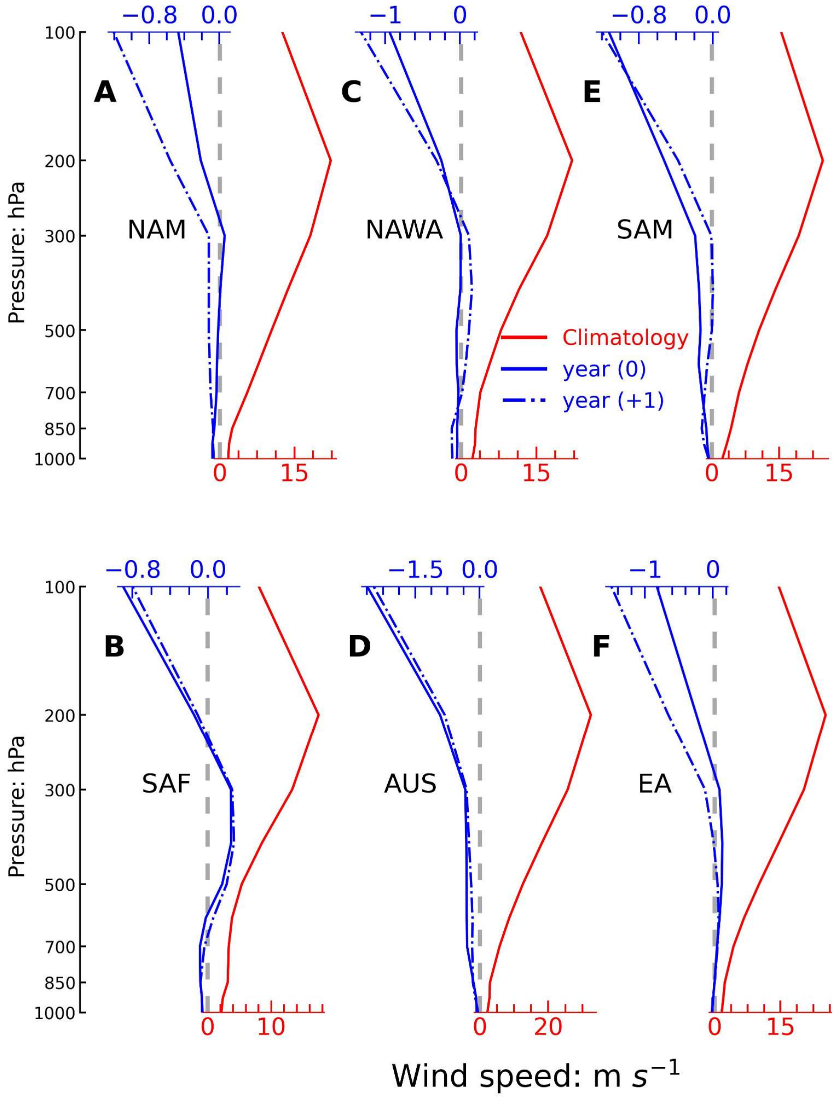

Wind speed over six subtropical regions in response to large tropical volcanic eruptions. (A) Annual-mean area-averaged wind speed (m s^{-1}) over North America (NAM) based on last-millennium simulations. Red solid, blue solid, and blue dotted lines denote climatology (five-year average before the eruption), anomalies in the eruption year, and anomalies in the first post-eruption year, respectively. (B–F) Same as (A), but for South Africa (SAF), North Africa–West Asia (NAWA), Australia (AUS), South America (SAM), and East Asia (EA) respectively.

Fig. S10.

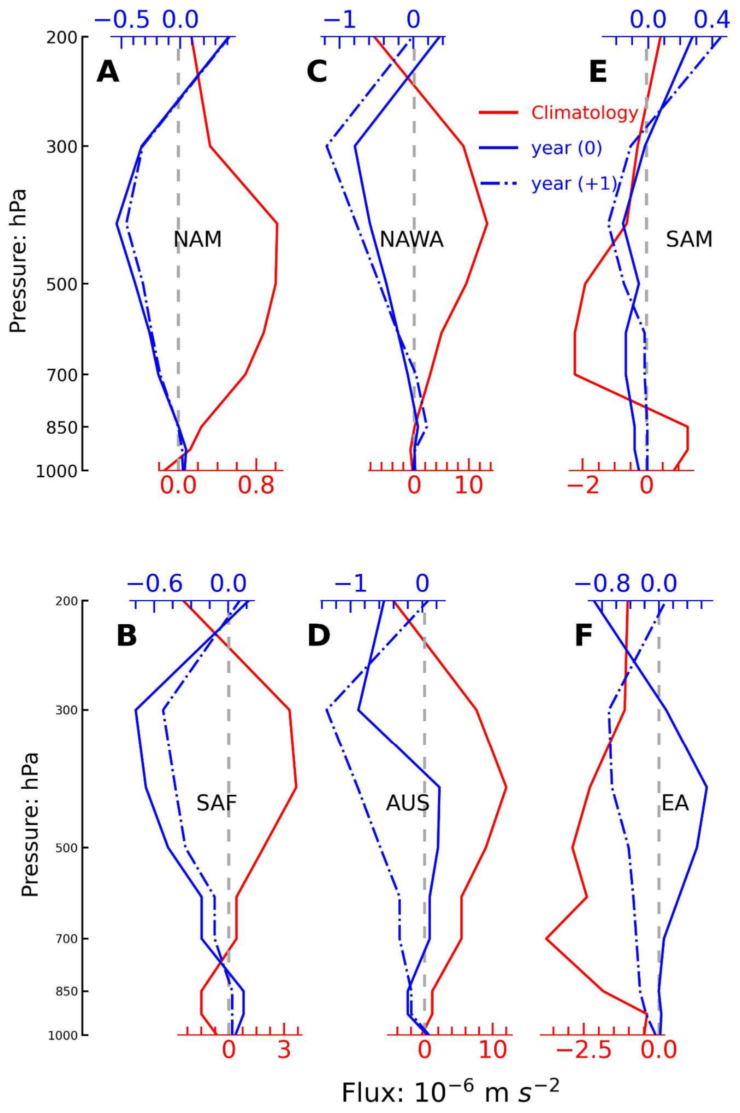

Vertical flux of horizontal momentum over six subtropical regions in response to large tropical volcanic eruptions. (A) Annual-mean area-averaged vertical flux of horizontal momentum ($10^6 \times$ m s^{-2}) over North America (NAM) based on last-millennium simulations. Red solid, blue solid, and blue dotted lines denote climatology (five-year average before the eruption), anomalies in the eruption year, and anomalies in the first post-eruption year, respectively. (B–F) Same as (A), but for South Africa (SAF), North Africa–West Asia (NAWA), Australia (AUS), South America (SAM), and East Asia (EA) respectively.
